# Cryptic Diversity in Indo-Australian Rainbowfishes Revealed by DNA Barcoding: Implications for Conservation in a Biodiversity Hotspot Candidate

**DOI:** 10.1371/journal.pone.0040627

**Published:** 2012-07-19

**Authors:** Kadarusman, Nicolas Hubert, Renny Kurnia Hadiaty, Sudarto, Emmanuel Paradis, Laurent Pouyaud

**Affiliations:** 1 Akademi Perikanan Sorong, Ministry of Marine Affairs and Fisheries, Sorong, West Papua, Indonesia; 2 UR226 ISE-M, Institut de Recherche pour le Développement, Montpellier, France; 3 Museum Zoologicum Bogoriense, Indonesian Institute of Sciences, Cibinong, West Java, Indonesia; 4 Institute for Research and Development of Ornamental Fish Culture, Ministry of Marine Affairs and Fisheries, Depok, West Java, Indonesia; University of Guelph, Canada

## Abstract

The rainbowfishes of the family *Melanotaeniidae* represent one of the largest radiations of freshwater fishes from the Indo-Australian archipelago. A total of 75 nominal species have been described, among which several have become very popular among tropical fish hobbyists because of their tendency to form large schools of colourful individuals. Facing habitat loss and competition or predation by introduced species, this group has become a priority in the conservation of ornamental fishes in Indonesia. In this context, several expeditions have been conducted between 2007 and 2010 in Indonesian Papua with the aim to initiate a large-scale survey of the genetic resources in this group. We assessed the diversity of the Papua rainbowfishes with DNA barcoding. We sequenced the mitochondrial COI gene for 350 specimens belonging to 53 nominal species throughout the Indo-Australian archipelago. Unexpected levels of cryptic diversity and endemism were detected since additional cryptic lineages were detected in several watersheds from the Vogelkop and the Lengguru massif. DNA barcoding supports the presence of nearly 30 evolutionary lineages among the 15 nominal species sampled in the Vogelkop and all these lineages are endemic to a single lake or watershed. This result highlights that the diversity of the family has been largely underestimated and urges for the identification of conservation priorities in Papua.

## Introduction

Species diversity is not evenly distributed on earth. Several regions, because they are exceptionally rich in endemic species and are facing massive habitat loss, have been identified as biodiversity hotspots [1]. Several terrestrial and marine biodiversity hotspots have been identified in the Indonesian archipelago as a consequence of unusual high levels of biodiversity threatened by increasing anthropogenic pressures [1], [2], [3]. In terrestrial biotas, two major hotspots have been identified on each side of the Wallace line: the Sundaland on the West including Borneo, Java, Sumatra and Bali, and Wallacea on the East including Sulawesi and Maluku [1]. Indonesian Papua and Papua New Guinea constitute New Guinea, the easternmost island of the Indonesian archipelago and currently one of the most poorly known regions of the world despite its proximity to remarkable biodiversity hotspots [4], [5], [6], [7].

**Figure 1:**
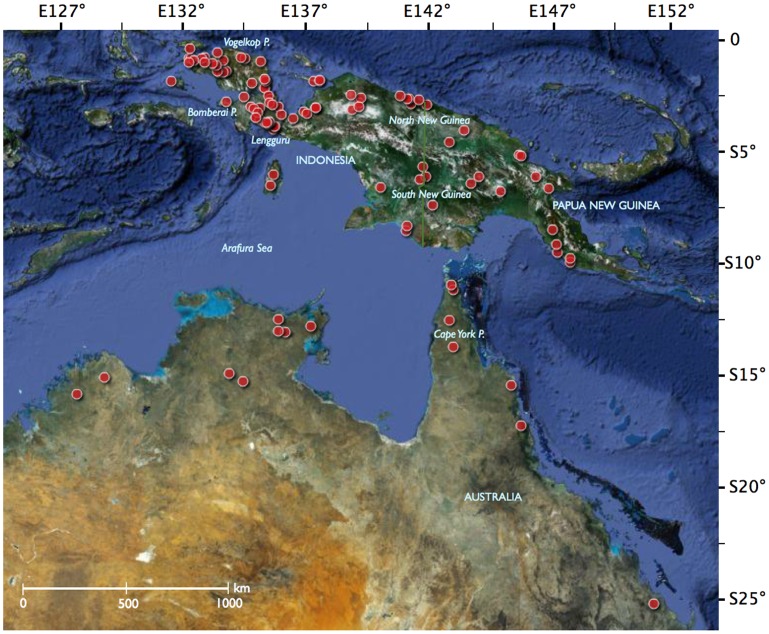
Map of the sampling localities.

Separated from Walacea by the Weber and Lydekker lines (Fig. 1), New Guinea is exceptionally rich in limestone karsts, some of the most inaccessible and understudied ecosystems in South East Asia [6], [7], [8], [9], [10]. Limestone karsts (karsts herein) are sedimentary outcrops mainly formed by reef-building corals in the past, erected above sea-level as a consequence of either tectonic activity or sea-level decrease and subsequently cleaned up from soft sediments through physical or chemical weathering [6]. These geological formations host a high diversity of species as a consequence of both a large array of ecological conditions due to complex landscapes from cliffs to subterranean structures and variable climatic conditions [9]. The presence of large karstic massifs is certainly one of the factors explaining why diversity and endemism is high in New Guinea [4], [5], [6], [7], [9]. At the same time, the anthropogenic pressure threatening karst biodiversity has significantly increased in South East Asia during the last decade as a consequence of mining [6], land burning for crop cultivation [11], logging activities [12], [13], and contamination [6].

The rainbowfishes of the family *Melanotaeniidae* provide a clear illustration of the biodiversity of New Guinea: (1) they represent one of the largest radiations of freshwater fishes from the Indo-Australian archipelago with 75 valid nominal species [14]; (2) a large number of species of the family are endemic to New Guinea where most species are confined to a single lake or tributary [4], [5], [14], [15], [16], [17], [18], [19]; (3) deforestation, mining activities and introduction of exotic species for aquaculture are increasing and constitute important threats to endemic species [5], [20]; and (4) several species of rainbowfishes are very popular among tropical fish hobbyists and catches in the field have dramatically increased during the last decade [14], [17].

Overall, species richness and number of endemic species are lower in New Guinea than in either Sundaland (Sumatra, Java, Borneo) or Wallacea (Sulawesi and Maluku) and, consequently, New Guinea has not been previously listed as a biodiversity hotspot candidate [1]. Given the deficit of biological prospection during the second half of the twentieth century compared to Borneo or Java [21], the number of endemic species may be expected to be underestimated and comparable to those observed elsewhere in the Indonesian archipelago. Considering the proliferation of threats on the ecosystems of New Guinea, extensive biological inventories are urgently needed to achieve conservation plans and identify priorities [22]. DNA barcoding, the use of a standardized molecular tag located in the mitochondrial cytochrome oxydase I gene for the identification of species [23], [24], has proven to be an effective tool for detecting cryptic diversity [25], [26], [27], [28]. In this context, we DNA-barcoded 350 specimens assigned with morphological characters to 53 nominal species of the families Melanotaeniidae and Atherinidae. All specimens were either collected during several expeditions conducted between 2007 and 2010 in Indonesian Papua, or obtained through the ‘Internationale Regenbogenfisch-Gesellschaft’ (IRG, the German association of rainbowfish hobbyists), which has successfully bred and maintained in captivity some referenced strains from New Guinea and Australia for several years. Overall, 15 among the 68 lineages found in the present study correspond to new and independent barcode clusters suggesting that both species diversity and endemism levels of the rainbowfishes of Papua are largely underestimated. Some implications for the conservation of the Papua freshwater biotas are discussed.

## Materials and Methods

Four expeditions were conducted between 2007 and 2010 in Indonesian Papua with the aim to conduct a large-scale survey of the rainbowfishes diversity through an integrative approach including barcoding as a primer step. A total of 350 specimens were barcoded. The samples came from two distinct sources: (1) the four above-mentioned expeditions were conducted by the French Institut de Recherche pour le Développement (IRD), the Indonesian Agency of Marine and Fisheries Research and Development (AMFRAD), the Indonesian Institute of Sciences (LIPI), and the Fisheries Academy of Sorong (Akademi Perikanan Sorong, APS) in the framework of a collaborative effort on the domestication and conservation of Indonesian rainbowfishes; (2) several samples were obtained through the IRG. In order to insure that the newly sampled populations are not representatives of some Australian lineages, 29 Australian rainbowfish species were included in our analysis. All the Australian species were obtained through the IRG. Likewise, the four above-mentioned institutions (IRD, AMAFRAD, LIPI and APS) approved all the expeditions and the present study. For each specimen, detailed geographic information and photographs were recorded, and reference specimens were deposited as vouchers in publicly available collections: the Museum Zoologicum Bogoriense in Bogor (MZB, Indonesia), the Naturalis Museum in Leiden (RMNH, Netherlands), and the Muséum National d’Histoire Naturelle in Paris (MNHN, France). Identifications were done by several of the present authors based on morphological criteria (colour, meristic counts) currently recognized in recent monographs and available data on the species ranges.

Genomic DNA was extracted using the Genomic DNA mini Kit (Tissue) of Geneaid Ltd according to manufacturer specifications and further used with no dilution for amplification and sequencing. The complete cytochrome oxydase I gene (COI) was amplified (1800 bp) using the primers FMEL-5′TCTTAGTTAACAGCTAAGCGC3′ and RMEL-5′GCGTCTTGGAATCCTAGTTG3′ (present study). When amplifications failed using this set of primers, a 650-bp segment from the 5′ region was amplified using the alternative reverse primers RANG-5′GTTGCGGAGGTAAAATAGGC3′, RMUN-5′GTCGCGGAAGTGAAATAGGC3′, RVOG-5′GTTGCAGAGGTGAAATAGGC3′ and RCATH-5′TGTTGCAGAGGTAAAATAGGC3′ (present study). Double-stranded DNA amplifications were performed with the FastStart PCR Master kit from Roche Ltd in 26 µL total reaction volume containing 13 µL of 2× master mix, 2 µL of each primer (stock solution 10 µM), 5 µL of template DNA (250–1000 ng) and 4 µL of ddH_2_O. Amplifications were carried out with an initial denaturation step at 95°C for 3 min followed by a cycle of three steps (94°C for 1 min, 58°C for 2 min, 72°C for 2 min 30 sec) repeated 34 times and ended by a final extension at 72°C for 10 min. Both strands of purified PCR fragments were sequenced by GenoScreen (Lille, France) using BIGDYE 3.1 protocol on 3730 XL 96 capillary sequencers (Applied Biosystems) using the same primers as for amplifications.

All the sequences have been deposited in GenBank and BOLD [29]. Accession numbers for the barcodes, specimen and collection data, sequences, trace files and primer details are available within the ‘Barcoding Indonesian Fishes - part I. Rainbowfishes from Papua’ (BIFA) project file under the general container ‘Barcoding fish – FishBOL’ in BOLD (http://www.barcodinglife.org; Table S1). Sequence divergence was calculated using the Kimura 2-parameter (K2P) model and the mid-point rooted Neighbour-joining (NJ) tree of K2P distances was created to provide a graphic representation of the species divergence as implemented in the ‘Sequence Analysis’ module of BOLD (Fig. S1). The phylogenetic structure of rainbowfish lineages across the main geographic regions sampled here was assessed with the additive partitioning approach of Hardy and Senterre [30]. The index of phylogenetic community structure, denoted as ∏_ST_, was computed and its significance was tested through 10,000 permutations using the R package SpacodiR [31]. This index provides a standardized estimate of phylogenetic community structure: ∏_ST_ <0 in case of phylogenetic dispersion across communities (i.e., species present in the same locality tend to be phylogenetically distant), whereas ∏_ST_ >0 if communities are phylogenetically clustered (i.e., species present in the same locality tend to be more phylogenetically related than on average). Finally, several hypotheses on the substitution rates of mitochondrial genes were compared to our geology-based calibrations of substitution rate for COI using dated tectonic events in Papua (Fig. 1).

## Results

**Figure 2:**
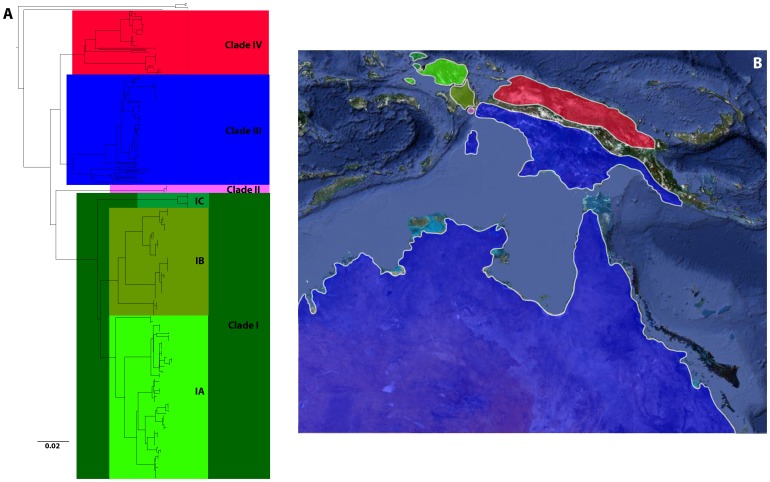
Major clades identified by COI barcodes and their geographic distribution. A, Neighbour-joining tree of 360 COI barcodes with the four major clades identified. B, Map of the four clade range distributions.

A 1800-bp PCR product for the complete COI gene was generated for 252 of the 350 individuals using the primer set FMEL and RMEL. Alternatively, a 650-bp PCR product for the 5′ half of the COI gene was obtained for the 98 remaining individuals using the reverse primers RANG (25 individuals), RCATH (11), RMUN (2) and RVOG (60). No insertions/deletions or codon stops were found as expected in protein-coding functional sequences, and thus the sequences were easily aligned. The full K2P-NJ tree is available as Supplementary Information (Fig. S1). A total of 350 barcodes were recovered for two families including one unidentified species from the genus *Craterocephalus* (Atherinidae) and 52 identified species from the family Melanotaeniidae belonging to the genera *Melanotaenia* (38 species), *Chilatherina* (5), *Glossolepis* (8) and *Iriatherina* (1). In addition, 14 new localities for *Melanotaenia* and one new locality for *Chilatherina* were discovered during the 2007–2010 expeditions (Table S1). Overall, four major clades are highlighted within the genera *Melanotaenia*, *Glossolepis* and *Chilatherina*, all being polyphyletic according to the COI sequences (Table S1; Fig. 1, 2 and S1). The first clade includes all *Melanotaenia* species from the Vogelkop beginning in the tree of Figure S1 with *M. arfakensis* and ending with *M. synergos*. The first clade is further divided into three sub-clades: IA from the main Vogelkop including from *M. arfakensis* to *M.* sp6, IB in the South Vogelkop (Bomberaï) from *M. irianjaya* to *M.* sp9, and IC in the Northen Raja Ampat from *M. catherinae* to *M. synergos* (Fig. 1, 2 and S1; Table S1). The second clade is monospecific and includes the very divergent *M. mairasi*, an endemic species from the Lengguru massif. The third clade includes all the *Melanotaenia* species from Australia and South New Guinea (from *M. pigmae* to *M. trifasciata* in Fig. S1). The fourth clade is restricted to North New Guinea and includes the genera *Glossolepis*, *Chilatherina* and part of *Melanotaenia* with *M. rubripinnis*, *M. affinis*, *M. maccullochi*, *M. praecox* and *M. vanheurni*. We assessed phylogenetic community structure across the seven main regions covered by the present study, Raja Ampat, Volgelkop, Bomberaï, Lengguru, South New Guinea, North New Guinea and Australia (Fig. 2), and found a highly significant pattern of phylogenetic clustering (∏_ST_  = 0.561, p-value <0.001). This result is consistent with the spatial structure depicted in Fig. 2.

Among the 52 species of Melanotaeniidae, the COI barcodes failed to capture species boundaries for 8 species of *Melanotaenia* and 3 species of *Glossolepis* whereas 30 species of *Melanotaenia*, 5 species of *Glossolepis* and the 5 species of *Chilatherina* exhibited non-overlapping COI clusters (Fig. S1). Mixed COI clusters were found between the populations of *Melanotaenia angfa* from Yakati and *M. parva*, among *M. kamaka*, *M. lakamora* and *M. pierucciae*, and between *M. rubripinnis* and *Glossolepis leggeti* from Wapoga (Fig. 1; Fig. S1). Interestingly, the populations found at the 14 new localities for *Melanotaenia* and the new locality for *Chilatherina* were all characterized by a private COI cluster suggesting their evolutionary distinctiveness from all the nominal species sampled in Papua (Fig. S1). Furthermore, these 15 new lineages were all distinct from the Australian species.

**Figure 3:**
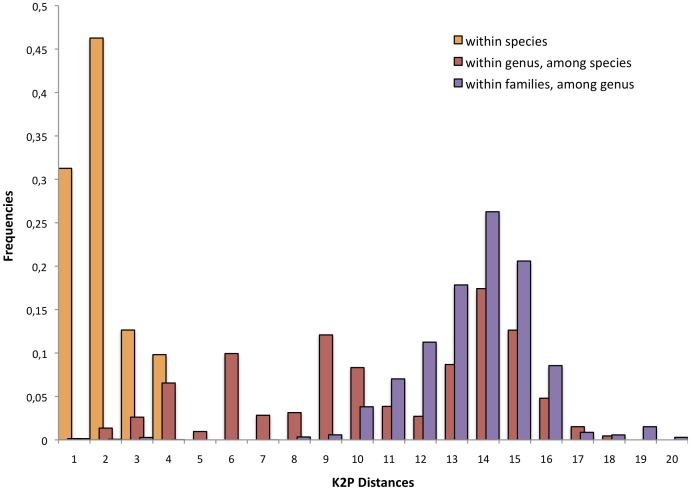
Distribution of the K2P distances within different taxonomic categories for the 350 individuals and 69 species analysed.

**Figure 4:**
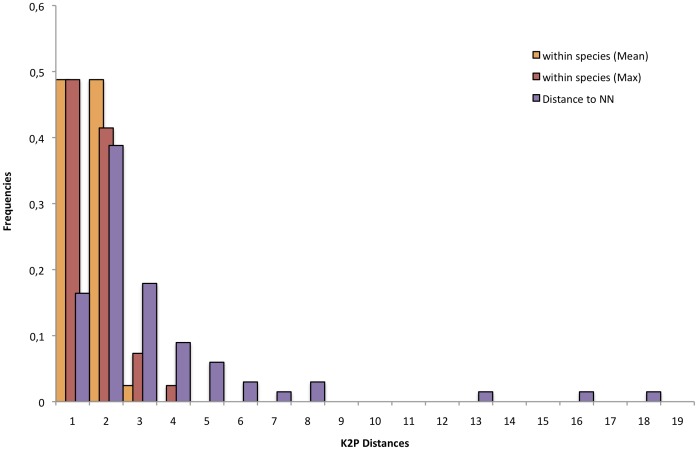
Distribution of the K2P distances to the nearest-neighbour, mean and maximum intra-specific distances.

When considering the 53 identified species and these 15 new lineages, a steady increase in genetic variation with increasing taxonomic levels was observed (Table 1; Fig. 3). Overall, genetic divergence among congeneric species was 14-fold higher on average than among individuals of the same species, a ratio twice smaller, however, than previously observed for fishes [32], [33], [34]. As a consequence of mixed COI clusters between some species, the minimum K2P distance among species was zero within genus (Table 1). More surprisingly, haplotype sharing between species was detected within family and between genera as the same haplotype was found in *M. rubripinnis* and *Glossolepis leggeti* leading to a minimum K2P distance of zero (Table 1). The distribution of the K2P distance to the nearest-neighbour shows that most sister-species diverge by K2P distances of less than 1% in either *Melanotaenia*, *Chilatherina*, or *Glossolepis* (Table 2; Fig. 4). When comparing this distribution among clades, only clade III harbours K2P distance to the nearest-neighbour higher than 1% in most of the species pairs examined (Table 2).

**Table 1:** Summary of K2P distances with respect to taxonomic levels.

Comparisons	n	taxa	Number of comparisons	Min	Mean	Max	SD
Within Species	322	41	2293	0	0.65	2.99	0.016
Within Genus, among species	349	4	43597	0	9.20	17.00	0.019
Within Family, among genus	350	2	13145	0	12.19	18.78	0.017
Within Order, among families	350	1	1725	16.566	19.64	22.22	0.020

Data are from 350 sequences from 69 species, five genera and two families (Table S1).

**Table 2:** Summary of the rainbowfish diversity analysed in this study and distribution of the genetic distance of each of the 66 species of Melanotaenidae (genera *Melanotaenia*, *Chilatherina* and *Glossolepis*) to the nearest-neighbour at COI (K2P model used for computing distances).

Taxa	Number of species	<0.1	0.1–1.0	1.0–2.7	>2.7
*Melanotaenia*	53	8	18	14	13
*Chilatherina*	6	0	4	1	1
*Glossolepis*	7	0	7	0	0
Clade IA	16	1	9	5	1
Clade IB	10	2	5	2	1
Clade III	22	5	2	7	8
Clade IV	18	5	9	1	3

The clades listed here correspond to the four major groups identified in the neighbour-joining tree (Fig. 2 and S1) and detailed in Table S1.

The initial divergence among Clades I, II, III and IV was estimated to have occurred during the Miocene between 27.4 and 21 Myr according to the ‘minimum’ substitution rate or between 6.9 and 5.3 Myr according to the canonical ‘vertebrate’ substitution rate (Table 3). The canonical ‘fish’ substitution rate provided an intermediate estimate between 11.4 and 8.8 Myr which is contemporaneous with an important stage of orogenic activity in the area that led to the uplift of the Lengguru arch between 12 and 9 Myr (Table 3). Likewise, calibrating the substitution rate with the uplift of the Lengguru arch provided an estimate of substitution rate between 0.010 and 0.017 substitutions per Myr, which is more in agreement with the canonical ‘fish’ rate than the two others.

## Discussion

The present DNA barcoding study has shown that the diversity of rainbowfishes in Papua is largely underestimated. This is particularly spectacular in the genus *Melanotaenia* where the 14 newly discovered populations exhibit private barcode clusters diverging from their nearest neighbor by K2P distances similar to those observed among valid species characterized by diagnostic morphological characters [14]. Altogether with the new lineage of *Chilatherina*, 15 new lineages of rainbowfishes are reported here. When compared with the 20 known species of *Melanotaenia* and *Chilatherina* sampled in West Papua during the present survey, DNA barcoding helped to highlight a cryptic diversity that almost multiply by two the number of known lineages in the area. By contrast, only 6 species among the 20 known species of *Melanotaenia*, *Chilatherina* and *Glossolepis* sampled in West Papua during the present study, exhibit complex boundaries that COI barcodes failed to capture (i.e., 13% of the 45 lineages sampled here). Thus, DNA barcoding was not only effective for the identification of species, but it proved to be effective for the discovery of provisional cryptic diversity awaiting further screening through integrative approaches as previously predicted by some authors [25], [26], [27], [28].

COI DNA barcoding has become an attractive approach for molecular identification due to the ease of amplification and sequencing of mitochondrial DNA, thanks to the large libraries of primers available and accessibility of this genome [35]. The maternal inheritance of this haploid genome, however, limits its power when applying to young species as a consequence of recent ancestry or hybridization that may prevent species delineation and identification if prior information is not available [36]. Hybridization has been previously reported in rainbowfishes including between species from distinct genera [17], [18], [19]. Here, only three cases of shared polymorphism were found between two species with mixed COI barcode clusters as *Melanotaenia angfa* (Lake Kurumoi) and *M. parva*, *M. kamaka* and *M. pierucciae* or *M. rubripinnis* and *Glossolepis leggetti* in the Wapoga River. Hybridization between *Melanotaenia* and *Glossolepis* is not unlikely since *Melanotaenia* is not monophyletic and encompass several lineages with at least two species (i.e., *M. rubripinnis* and *M. affinis*) more closely related to *Glossolepis* species than other *Melanotaenia* species. Interestingly, inter-generic hybridization has been previously reported between *Melanotaenia* and *Chilatherina* [17] and the presence of haplotype sharing makes the hypothesis of hybridization and introgression of mitochondrial DNA very likely [36]. These limitations, however, are unlikely to affect our estimation of the number of cryptic lineages since all the 15 new lineages identified here are spatially isolated and characterized by a private set of barcode cluster.

**Table 3:** Summary of the phylogenetic variability within and among the four major clades, clades age estimates according to three hypotheses of molecular calibration and calibrations of absolute substitution rates per millions years of COI sequences based on geology.

						Geological event age (Myr)		Calibration (substitution rates per Myr)	
	Mean	SE	H1^a^ (Myr)	H2^b^ (Myr)	H3^c^ (Myr)	Min	Max	Min	Max
within I	0.059	0.005	11.8	4.9	3.0	–	–	–	–
within IA	0.033	0.004	6.6	2.8	1.7	–	–	–	–
within IB	0.032	0.004	6.4	2.7	1.6	1.65^d^	3.4^d^	0.0194^f^	0.0094^f^
within III	0.042	0.004	8.4	3.5	2.1	–	–	–	–
within IV	0.051	0.006	10.2	4.3	2.6	–	–	–	–
I vs II	0.137	0.013	27.4	11.4	6.9	8^e^	11^e^	0.017	0.012
I vs III	0.128	0.012	25.6	10.7	6.4	8^e^	11^e^	0.016	0.012
I vs IV	0.130	0.011	26	10.8	6.5	8^e^	11^e^	0.016	0.012
II vs III	0.137	0.014	27.4	11.4	6.9	8^e^	11^e^	0.017	0.012
II vs IV	0.126	0.012	25.2	10.5	6.3	8^e^	11^e^	0.016	0.011
III vs IV	0.105	0.010	21	8.8	5.3	8^e^	11^e^	0.013	0.010
IA vs IB	0.077	0.009	15.4	6.4	3.9	–	–	–	–

a assuming a ‘minimum’ substitution rate of 0.005 substitution per Myr [38], [40].

b assuming a ‘fish’ substitution rate of 0.012 substitution per Myr [39].

c assuming a ‘vertebrate’ substitution rate of 0.02 substitution per Myr [38].

d calibration based on the mean K2P distance among species within clade IB and assuming that most speciation event in clade IB are related with the uplift of the Mok ridge [41], [42].

e estimated geological age for the uplift of the Lengguru arch [41], [42].

f substitution rate estimated based on the calibration with the geological age for the uplift of the Mok ridge.

The pattern of cryptic diversity detected here largely confirms the importance of New Guinea and Papua for the biodiversity of the rainbowfishes and shed new light on the potential influence of this area in their evolution [18], [19], [37]. First, this survey confirms that most of the phylogenetic diversity is found in Papua since the four clades identified here are present there. Second, in agreement with the comparisons made here between previously published hypotheses of calibration of substitution rates [38], [39], [40] and the present geology-based calibrations [41], [42], it is likely that the uplift of the Lengguru massif is at the origin of the first cladogenetic events in the genera *Melanotaenia*, *Chilatherina* and *Glossolepis* and the emergence of the clades I, II, III and IV (Fig. 1 and 2). This assumption is supported by the agreement of the substitution rate estimates obtained through the specific calibration of COI for fishes [39] and the geology-based estimates obtained here [41], [42], which points to an early diversification stage in the Melanotaeniidae crown group around 11 Myr contemporaneous with the uplift of the Lengguru arch between 11 and 8 Myr. Furthermore, Clade 2 is constituted by a single species, *Melanotaenia maerasi*, endemic to the Lake Kuweri, a watershed that has been isolated during the uplift of the Lengguru Arch. Quite interestingly, *M. maerasi* is estimated to have emerged around 11 Myr and constitutes the oldest rainbowfish species reported to date. Third, Clades I and II are the oldest clades according to our estimations as their divergence is estimated to have occured around 11.4 Myr while the eastern clades III and IV are estimated to have diverged around 8.8 Myr (Fig. 2; Table 3). Altogether these results suggest that Western Papua including the Vogelkop is a persistence of the centre of origin of the group that probably took place in this area during the last 10 Myr, a result which has been previously suggested [18], [19] but needed a thorough molecular survey in West Papua.

The present study provides also some new insights on the evolution of biodiversity in New Guinea since the high number of lineages detected here, belonging to a single river or lake, confirms that karsts are highly fragmented landscapes which foster geographic isolation and promote endemism [4], [5], [6], [7]. Endemism levels are likely to be underestimated in Papua, however, as a consequence of the complexity of karstic landscapes and the remoteness of the area. Our results suggest that diversity might be twice as high as previously thought and endemism dominate in karstic communities. Yet, the estimated freshwater fish diversity and endemism of New Guinea freshwater fishes is lower than that observed in Borneo, Sumatra or Java which exhibit twice as many species [43]. Given the threats on their biodiversity, Borneo, Sumatra and Java have been identified as a biodiversity hotspot and a priority for conservation [1]. Our results suggest, however, that the number of endemic species may be actually higher in New Guinea than elsewhere in the Indonesian archipelago and that there might be no objective reasons to not consider New Guinea as a biodiversity hotspot for freshwaters in the future.

Several human activities are currently threatening natural ecosystems in New Guinea including logging, oil and gas concessions, mining activities, land burning for crop cultivation and development of new roads [44], [45]. Altogether, these threats have conducted to the physical degradation and contamination of the New Guinea inland water ecosystems further threatened by the harvesting of natural resources including fisheries and ornamental trade but also the introduction of exotic species [4], [9], [15], [20]. Many rainbowfish species are tightly restricted to single lakes or small watersheds and as such, they are highly vulnerable to environmental disturbances [15], [17], [37]. As a consequence, 7 species of the genus *Melanotaenia* from the 20 species reported here from Papua are already considered threatened according to Conservation International 2002 and IUCN 2009. Conservation of the rainbowfishes biodiversity is challenging, however, as reproductive compatibilities among species seem to be maintained long time after speciation leading to ease of hybridization even among inter-generic species [18], [19], [37]. The great diversity of phenotypes found in the family, which is at the origin of its success in the aquarium trade, however, is tightly linked to the maintenance of their genetic diversity as previously reported for other emblematic species of the aquarium fish trade [46], [47]. As a consequence, conservation programs implying breeding plans should pay attention to hybridization in order to prevent the loss of phenotypic diversity or alter fitness in natural populations through translocation programs. Considering the large amount of cryptic diversity discovered here, this is undoubtedly a challenging task.

## Supporting Information

Figure S1 **Neighbour-joining tree of 360 COI barcodes belonging to the 69 species examined here and extracted from BOLD.**
(PDF)

Table S1 **Details of species and specimens.** Barcode of Life Database (BOLD) specimen numbers given, along with GenBank accession numbers, geographic locality and voucher details. Assignation of each species and specimens to one of the four major clades according to the neighbour-joining tree (Fig. S1) are indicated here.
(XLS)

## References

[1] Myers N, Mittermeier RA, Mittermeier CG, da Fonseca GAB, Kent J (2002). Biodiversity hotspots for conservation priorities.. Nature.

[2] Roberts CM, McClean CJ, Veron JEN, Hawkins JP, Allen GR (2002). Marine biodiversity hotspots and conservation priorities for tropical reefs.. Science.

[3] Mora C, Chittaro PM, Sale PF, Kritzer JP, Ludsin SA (2003). Patterns and processes in reef fish diversity.. Nature.

[4] Polhemus DA, Allen GR, Marshall AJ, Beehler BM (2006). Freshwater biogeography of Papua..

[5] Polhemus DA, Englund RA, Allen GR (2004). Freshwater biotas of new guinea and nearby islands: analysis of endemism, richness, and threats.. 68 p.

[6] Clements R, Sodhi NS, Schilthuizen M, Ng PKL (2006). Limestone karsts of Southeast Asia: imperiled arks of biodiversity.. Bioscience.

[7] Clements R, Ng PKL, Lu XX, Ambu S, Schilthuizen M (2008). Using biogeographical patterns of endemic land snails to improve conservation planning for limestone karsts.. Biological Conservation.

[8] Dennis C, Aldhous P (2004). Biodiversity: a tragedy with many players.. Nature.

[9] Marshall AJ, Marshall AJ, Beehler BM (2006). The diversity and conservation of Papua’s ecosystems..

[10] Marshall AJ, Beehler BM (2006). The ecology of Papua.. Singapore: Periplus Editions.

[11] Schilthuizen M, Liew TS, Bin Elehan B, Lackman-Ancrenaz I (2005). Effects of karst forest degradation on pulmonate and prosobranch land snail communities in Sabah, Malaysian Borneo.. Conservation Biology.

[12] Kiew R, Kiew R (1991). The limestone flora..

[13] Culver DC, Master LL, Christman MC, Hobbs HH (2000). Obligate cave fauna of the 48 contiguous united states.. Conservation Biology.

[14] Tappin AR (2011). Rainbowfishes - Their care & keeping in captivity.. Second edition: Art publications.

[15] Allen GR, Marshall AJ, Beehler BM (2006). Fishes of Papua..

[16] Froese R, Pauly D (2011). Fishbase.. http://www.fishbase.org.

[17] Allen GR, Cross NJ (1982). Rainbowfishes of Australia and Papua New Guinea.. New Jersey: T.F.H. Publications.

[18] McGuigan K, Zhu D, Allen GR, Moritz C (2000). Phylogenetic relationships and historical biogeography of melanotaeniid fishes in Australia and New Guinea.. Marine & Freshwater Research.

[19] Zhu D, Jamieson A, Hugall A, Moritz C (1994). Sequence evolution and phylogenetic signal in control-region and cytochrome b sequences of rainbowfishes (Melanotaeniidae).. Molecular Biology and Evolution.

[20] Fraser S, Marshall AJ, Beehler BM (2006). Threats to biodiversity..

[21] Frodin DG, Marshall AJ, Beehler BM (2006). Biological exploration of New Guinea..

[22] Burnett JB, Marshall AJ, Beehler BM (2006). Setting priorities and planning conservation in Papua..

[23] Hebert PDN, Cywinska A, Ball SL, de WaardJR (2003). Biological identifications through DNA barcodes.. Proceedings of the Royal Society of London Series B.

[24] Hebert PDN, Gregory TR (2005). The promise of DNA barcoding for taxonomy.. Systematic Biology.

[25] Hebert PDN, Penton EH, Burns JM, Janzen DH, Hallwachs W (2004). Ten species in one: DNA barcoding reveals cryptic species in the neotropical skipper butterfly Astraptes fulgerator.. Proceedings of the National Academy of Sciences, USA.

[26] Smith AM, Rodriguez JJ, Whitfield JB, Deans AR, Janzen DH (2008). Extreme diversity of tropical parasitoid wasps exposed by iterative integration of natural history, DNA barcoding, morphology, and collections.. Proceedings of the National Academy of Sciences, USA.

[27] Smith MA, Wood DM, Janzen DH, Hallwachs W, Hebert PDN (2007). DNA barcodes affirm that 16 species of apparently generalist tropical parasitoid flies (Diptera, Tachinidae) are not all generalists.. Proceedings of the National Academy of Sciences, USA.

[28] Hubert N, Meyer C, Bruggemann JH, Guérin F, Komeno RJL (2012). Cryptic diversity in Indo-Pacific coral reef fishes revealed by DNA-barcoding provides new support to the centre-of-overlap hypothesis.. PLoS one.

[29] Ratnasingham S, Hebert PDN (2007). Molecular ecology notes. http://www.barcodinglife.org.

[30] Hardy OJ, Senterre B (2007). Characterizing the phylogenetic structure of communities by an additive partitioning of phylogenetic diversity.. Journal of Ecology.

[31] Eastman JM, Paine CET, Hardy OJ (2011). spacodiR: structuring of phylogenetic diversity in ecological commuities.. Bioinformatics.

[32] Hubert N, Hanner RH, Holm E, Mandrak NE, Taylor EB (2008). Identifying Canadian freshwater fishes through DNA barcodes.. PLoS one.

[33] Ward RD, Zemlak TS, Innes BH, Last PR, Hebert PDN (2005). DNA barcoding Australia’s fish species.. Phylosophical Transactions of the Royal Society B.

[34] April J, Mayden RL, Hanner RH, Bernatchez L (2011). Genetic calibration of species diversity among North America’s freshwater fishes.. Proceedings of the National Academy of Sciences, USA.

[35] Schindel D, Miller SE (2005). DNA barcoding, a useful tool for taxonomist.. Nature.

[36] Funk DJ, Omland KE (2003). Species-level paraphyly and polyphyly: frequency, causes and consequences, with insights from animal mitochondrial DNA.. Annual Review of Ecology and Systematic.

[37] Allen GR (1995). Rainbowfishes in nature and the aquarium.. Melle, Germany: Tetra Publications.

[38] Read CI, Bellwood DR, Herwerden van L (2006). Ancient origins of Indo-Pacific coral reef fish biodiversity: a case study of the Leopard wrasses (Labridae: Macropharyngodon).. Molecular Phylogenetics and Evolution.

[39] Bermingham E, McCafferty S, Martin AP, Kocher TD, Stepien CA (1997). Fish biogeography and molecular clocks: perspectives from the Panamanian isthmus..

[40] Hardman M, Lundberg JG (2006). Molecular phylogeny and chronology of diversification for “phractocephaline” catfishes (Siluriformes: Pimelodidae) based on mitochondrial DNA and DNA recombination activating gene 2 sequences.. Molecular Phylogenetics and Evolution.

[41] Villeneuve M, Martini R, Bellon H, Réhault J-P, Cornée J-J (2010). Deciphering of six blocks of Gondwana origin within Eastern Indonesia (South East Asia).. Gondwana Research.

[42] Bailly V, Pubellier M, Ringenbach JC, de Sigoyer J, Sapin F (2009). Deformation zone ‘jumps’ in a young convergent setting; the Lengguru fold-and-thrust belt, New Guinea Island.. Lithos.

[43] Abell R, Thieme ML, Revenga C, Bryer M, Kottelat M (2008). Freshwater ecoregions of the world: A new map of biogeographic units for freshwater biodiversity conservation.. Bioscience.

[44] Mertens B (2002). Spatial analyses for the rapid assessment of conservation and economy (RACE) in Papua.. Centre for International Forestry Research.

[45] McAlpine JR, Freyne DF (2001). Land use change and intensificarion in Papua New Guinea 1975–1996.. Asia PAcific Viewpoint.

[46] Steinke D, Zemlak TS, Hebert PDN (2009). Barcoding Nemo: DNA-based identifications for the ornamental fish trade.. PLoS one.

[47] Sriwattanarothai N, Steinke D, Ruenwongsa P, Hanner R, Panijpan B (2010). Molecular and morphological evidence supports the species status of the mahachai fighter (Betta sp. mahachai) and reveals new species of Betta from Thailand.. Journal of Fish Biology.

