# Supplementary material for: Cryptic Diversity in Indo-Australian Rainbowfishes Revealed by DNA Barcoding: Implications for Conservation in a Biodiversity Hotspot Candidate
Source: PLoS One. 2012 Jul 19;7(7):e40627. doi: 10.1371/journal.pone.0040627 (PMC3400673; doi:10.1371/journal.pone.0040627)
Supplement: Figure S1 — Neighbour-joining tree of 360 COI barcodes belonging to the 69 species examined here and extracted from BOLD. (PDF) [file pone.0040627.s001.pdf]

BOLD TaxonID Tree

```
Project         : Barcoding Indonesian Fishes - part I. Rainbowfishes from Pap...
Date            : 21-October-2011
Data Type       : Nucleotide
Distance Model  : Kimura 2 Parameter
Marker          : COI-5P
Codon Positions : 1st, 2nd, 3rd
Labels          : SampleID,
Filters         : Length > 200
Colorization    : [blue]=Stop Codons [red]=Contamination or misidentification

Sequence Count  : 350
Species count   : 67
Genus count     : 5
Family count    : 2
Unidentified    : 2
```

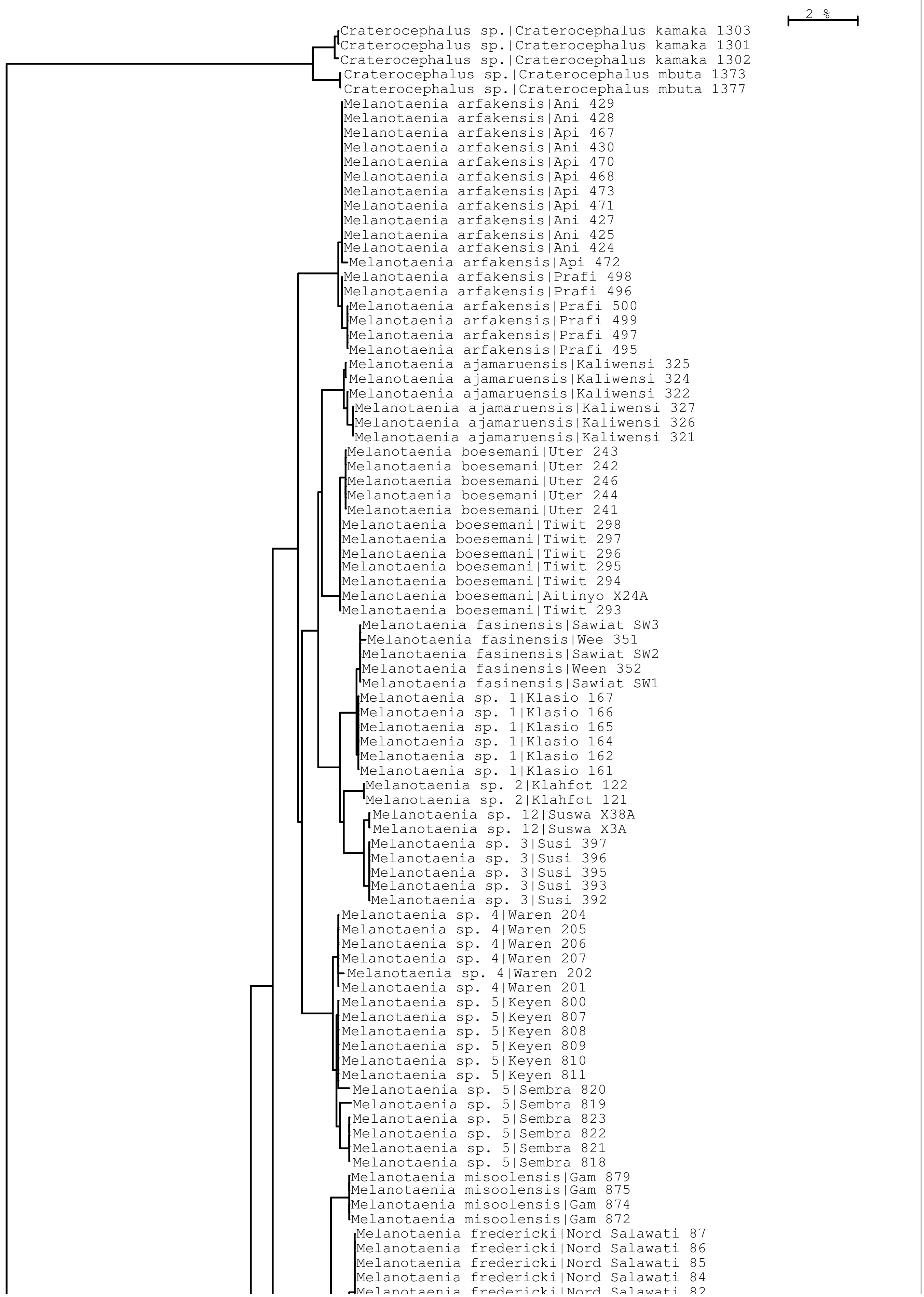
2 %
Craterocephalus sp.|Craterocephalus kamaka 1303
Craterocephalus sp.|Craterocephalus kamaka 1301
Craterocephalus sp.|Craterocephalus kamaka 1302
Craterocephalus sp.|Craterocephalus mbuta 1373
Craterocephalus sp.|Craterocephalus mbuta 1377
Melanotaenia arfakensis|Ani 429
Melanotaenia arfakensis|Ani 428
Melanotaenia arfakensis|Api 467
Melanotaenia arfakensis|Ani 430
Melanotaenia arfakensis|Api 470
Melanotaenia arfakensis|Api 468
Melanotaenia arfakensis|Api 473
Melanotaenia arfakensis|Api 471
Melanotaenia arfakensis|Ani 427
Melanotaenia arfakensis|Ani 425
Melanotaenia arfakensis|Ani 424
Melanotaenia arfakensis|Api 472
Melanotaenia arfakensis|Prafi 498
Melanotaenia arfakensis|Prafi 496
Melanotaenia arfakensis|Prafi 500
Melanotaenia arfakensis|Prafi 499
Melanotaenia arfakensis|Prafi 497
Melanotaenia arfakensis|Prafi 495
Melanotaenia ajamaruensis|Kaliwensi 325
Melanotaenia ajamaruensis|Kaliwensi 324
Melanotaenia ajamaruensis|Kaliwensi 322
Melanotaenia ajamaruensis|Kaliwensi 327
Melanotaenia ajamaruensis|Kaliwensi 326
Melanotaenia ajamaruensis|Kaliwensi 321
Melanotaenia boesemani|Uter 243
Melanotaenia boesemani|Uter 242
Melanotaenia boesemani|Uter 246
Melanotaenia boesemani|Uter 244
Melanotaenia boesemani|Uter 241
Melanotaenia boesemani|Tiwit 298
Melanotaenia boesemani|Tiwit 297
Melanotaenia boesemani|Tiwit 296
Melanotaenia boesemani|Tiwit 295
Melanotaenia boesemani|Tiwit 294
Melanotaenia boesemani|Aitinyo X24A
Melanotaenia boesemani|Tiwit 293
Melanotaenia fasinensis|Sawiat SW3
Melanotaenia fasinensis|Wee 351
Melanotaenia fasinensis|Sawiat SW2
Melanotaenia fasinensis|Ween 352
Melanotaenia fasinensis|Sawiat SW1
Melanotaenia sp. 1|Klasio 167
Melanotaenia sp. 1|Klasio 166
Melanotaenia sp. 1|Klasio 165
Melanotaenia sp. 1|Klasio 164
Melanotaenia sp. 1|Klasio 162
Melanotaenia sp. 1|Klasio 161
Melanotaenia sp. 2|Klahfot 122
Melanotaenia sp. 2|Klahfot 121
Melanotaenia sp. 12|Suswa X38A
Melanotaenia sp. 12|Suswa X3A
Melanotaenia sp. 3|Susi 397
Melanotaenia sp. 3|Susi 396
Melanotaenia sp. 3|Susi 395
Melanotaenia sp. 3|Susi 393
Melanotaenia sp. 3|Susi 392
Melanotaenia sp. 4|Waren 204
Melanotaenia sp. 4|Waren 205
Melanotaenia sp. 4|Waren 206
Melanotaenia sp. 4|Waren 207
Melanotaenia sp. 4|Waren 202
Melanotaenia sp. 4|Waren 201
Melanotaenia sp. 5|Keyen 800
Melanotaenia sp. 5|Keyen 807
Melanotaenia sp. 5|Keyen 808
Melanotaenia sp. 5|Keyen 809
Melanotaenia sp. 5|Keyen 810
Melanotaenia sp. 5|Keyen 811
Melanotaenia sp. 5|Sembra 820
Melanotaenia sp. 5|Sembra 819
Melanotaenia sp. 5|Sembra 823
Melanotaenia sp. 5|Sembra 822
Melanotaenia sp. 5|Sembra 821
Melanotaenia sp. 5|Sembra 818
Melanotaenia misoolensis|Gam 879
Melanotaenia misoolensis|Gam 875
Melanotaenia misoolensis|Gam 874
Melanotaenia misoolensis|Gam 872
Melanotaenia fredericki|Nord Salawati 87
Melanotaenia fredericki|Nord Salawati 86
Melanotaenia fredericki|Nord Salawati 85
Melanotaenia fredericki|Nord Salawati 84
Melanotaenia fredericki|Nord Salawati 82

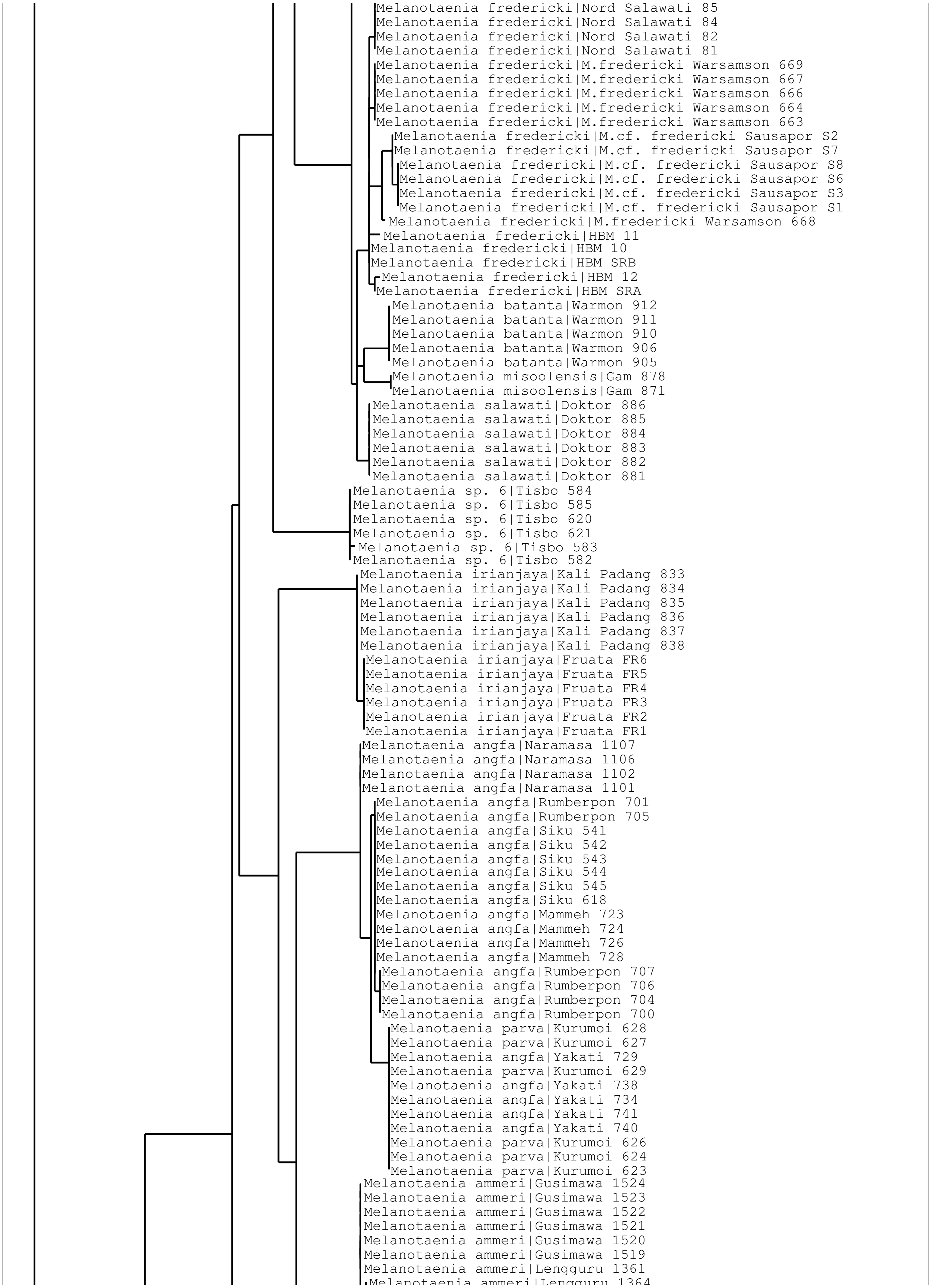
Melanotaenia fredericki|Nord Salawati 85
Melanotaenia fredericki|Nord Salawati 84
Melanotaenia fredericki|Nord Salawati 82
Melanotaenia fredericki|Nord Salawati 81
Melanotaenia fredericki|M.fredericki Warsamson 669
Melanotaenia fredericki|M.fredericki Warsamson 667
Melanotaenia fredericki|M.fredericki Warsamson 666
Melanotaenia fredericki|M.fredericki Warsamson 664
Melanotaenia fredericki|M.fredericki Warsamson 663
Melanotaenia fredericki|M.cf. fredericki Sausapor S2
Melanotaenia fredericki|M.cf. fredericki Sausapor S7
Melanotaenia fredericki|M.cf. fredericki Sausapor S8
Melanotaenia fredericki|M.cf. fredericki Sausapor S6
Melanotaenia fredericki|M.cf. fredericki Sausapor S3
Melanotaenia fredericki|M.cf. fredericki Sausapor S1
Melanotaenia fredericki|M.fredericki Warsamson 668
Melanotaenia fredericki|HBM 11
Melanotaenia fredericki|HBM 10
Melanotaenia fredericki|HBM SRB
Melanotaenia fredericki|HBM 12
Melanotaenia fredericki|HBM SRA
Melanotaenia batanta|Warmon 912
Melanotaenia batanta|Warmon 911
Melanotaenia batanta|Warmon 910
Melanotaenia batanta|Warmon 906
Melanotaenia batanta|Warmon 905
Melanotaenia misoolensis|Gam 878
Melanotaenia misoolensis|Gam 871
Melanotaenia salawati|Doktor 886
Melanotaenia salawati|Doktor 885
Melanotaenia salawati|Doktor 884
Melanotaenia salawati|Doktor 883
Melanotaenia salawati|Doktor 882
Melanotaenia salawati|Doktor 881
Melanotaenia sp. 6|Tisbo 584
Melanotaenia sp. 6|Tisbo 585
Melanotaenia sp. 6|Tisbo 620
Melanotaenia sp. 6|Tisbo 621
Melanotaenia sp. 6|Tisbo 583
Melanotaenia sp. 6|Tisbo 582
Melanotaenia irianjaya|Kali Padang 833
Melanotaenia irianjaya|Kali Padang 834
Melanotaenia irianjaya|Kali Padang 835
Melanotaenia irianjaya|Kali Padang 836
Melanotaenia irianjaya|Kali Padang 837
Melanotaenia irianjaya|Kali Padang 838
Melanotaenia irianjaya|Fruata FR6
Melanotaenia irianjaya|Fruata FR5
Melanotaenia irianjaya|Fruata FR4
Melanotaenia irianjaya|Fruata FR3
Melanotaenia irianjaya|Fruata FR2
Melanotaenia irianjaya|Fruata FR1
Melanotaenia angfa|Naramasa 1107
Melanotaenia angfa|Naramasa 1106
Melanotaenia angfa|Naramasa 1102
Melanotaenia angfa|Naramasa 1101
Melanotaenia angfa|Rumberpon 701
Melanotaenia angfa|Rumberpon 705
Melanotaenia angfa|Siku 541
Melanotaenia angfa|Siku 542
Melanotaenia angfa|Siku 543
Melanotaenia angfa|Siku 544
Melanotaenia angfa|Siku 545
Melanotaenia angfa|Siku 618
Melanotaenia angfa|Mammeh 723
Melanotaenia angfa|Mammeh 724
Melanotaenia angfa|Mammeh 726
Melanotaenia angfa|Mammeh 728
Melanotaenia angfa|Rumberpon 707
Melanotaenia angfa|Rumberpon 706
Melanotaenia angfa|Rumberpon 704
Melanotaenia angfa|Rumberpon 700
Melanotaenia parva|Kurumoi 628
Melanotaenia parva|Kurumoi 627
Melanotaenia angfa|Yakati 729
Melanotaenia parva|Kurumoi 629
Melanotaenia angfa|Yakati 738
Melanotaenia angfa|Yakati 734
Melanotaenia angfa|Yakati 741
Melanotaenia angfa|Yakati 740
Melanotaenia parva|Kurumoi 626
Melanotaenia parva|Kurumoi 624
Melanotaenia parva|Kurumoi 623
Melanotaenia ammeri|Gusimawa 1524
Melanotaenia ammeri|Gusimawa 1523
Melanotaenia ammeri|Gusimawa 1522
Melanotaenia ammeri|Gusimawa 1521
Melanotaenia ammeri|Gusimawa 1520
Melanotaenia ammeri|Gusimawa 1519
Melanotaenia ammeri|Lengguru 1361
Melanotaenia ammeri|Lengguru 1364

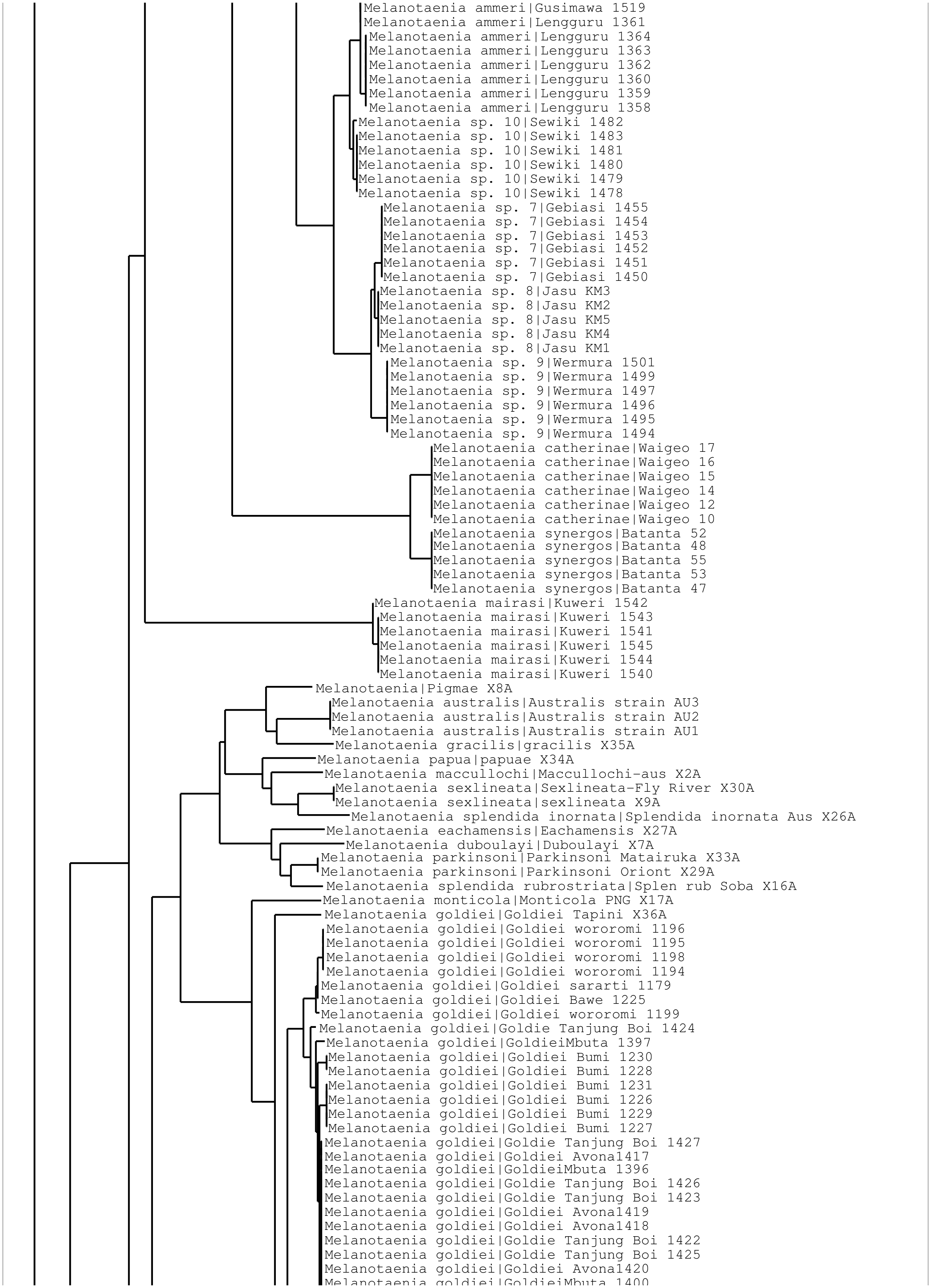
Melanotaenia ammeri|Gusimawa 1519
Melanotaenia ammeri|Lengguru 1361
Melanotaenia ammeri|Lengguru 1364
Melanotaenia ammeri|Lengguru 1363
Melanotaenia ammeri|Lengguru 1362
Melanotaenia ammeri|Lengguru 1360
Melanotaenia ammeri|Lengguru 1359
Melanotaenia ammeri|Lengguru 1358
Melanotaenia sp. 10|Sewiki 1482
Melanotaenia sp. 10|Sewiki 1483
Melanotaenia sp. 10|Sewiki 1481
Melanotaenia sp. 10|Sewiki 1480
Melanotaenia sp. 10|Sewiki 1479
Melanotaenia sp. 10|Sewiki 1478
Melanotaenia sp. 7|Gebiasi 1455
Melanotaenia sp. 7|Gebiasi 1454
Melanotaenia sp. 7|Gebiasi 1453
Melanotaenia sp. 7|Gebiasi 1452
Melanotaenia sp. 7|Gebiasi 1451
Melanotaenia sp. 7|Gebiasi 1450
Melanotaenia sp. 8|Jasu KM3
Melanotaenia sp. 8|Jasu KM2
Melanotaenia sp. 8|Jasu KM5
Melanotaenia sp. 8|Jasu KM4
Melanotaenia sp. 8|Jasu KM1
Melanotaenia sp. 9|Wermura 1501
Melanotaenia sp. 9|Wermura 1499
Melanotaenia sp. 9|Wermura 1497
Melanotaenia sp. 9|Wermura 1496
Melanotaenia sp. 9|Wermura 1495
Melanotaenia sp. 9|Wermura 1494
Melanotaenia catherinae|Waigeo 17
Melanotaenia catherinae|Waigeo 16
Melanotaenia catherinae|Waigeo 15
Melanotaenia catherinae|Waigeo 14
Melanotaenia catherinae|Waigeo 12
Melanotaenia catherinae|Waigeo 10
Melanotaenia synergos|Batanta 52
Melanotaenia synergos|Batanta 48
Melanotaenia synergos|Batanta 55
Melanotaenia synergos|Batanta 53
Melanotaenia synergos|Batanta 47
Melanotaenia mairasi|Kuweri 1542
Melanotaenia mairasi|Kuweri 1543
Melanotaenia mairasi|Kuweri 1541
Melanotaenia mairasi|Kuweri 1545
Melanotaenia mairasi|Kuweri 1544
Melanotaenia mairasi|Kuweri 1540
Melanotaenia|Pigmae X8A
Melanotaenia australis|Australis strain AU3
Melanotaenia australis|Australis strain AU2
Melanotaenia australis|Australis strain AU1
Melanotaenia gracilis|gracilis X35A
Melanotaenia papua|papuae X34A
Melanotaenia maccullochi|Maccullochi-aus X2A
Melanotaenia sexlineata|Sexlineata-Fly River X30A
Melanotaenia sexlineata|sexlineata X9A
Melanotaenia splendida inornata|Splendida inornata Aus X26A
Melanotaenia eachamensis|Eachamensis X27A
Melanotaenia duboulayi|Duboulayi X7A
Melanotaenia parkinsoni|Parkinsoni Matairuka X33A
Melanotaenia parkinsoni|Parkinsoni Oriont X29A
Melanotaenia splendida rubrostriata|Splen rub Soba X16A
Melanotaenia monticola|Monticola PNG X17A
Melanotaenia goldiei|Goldiei Tapini X36A
Melanotaenia goldiei|Goldiei wororomi 1196
Melanotaenia goldiei|Goldiei wororomi 1195
Melanotaenia goldiei|Goldiei wororomi 1198
Melanotaenia goldiei|Goldiei wororomi 1194
Melanotaenia goldiei|Goldiei sararti 1179
Melanotaenia goldiei|Goldiei Bawe 1225
Melanotaenia goldiei|Goldiei wororomi 1199
Melanotaenia goldiei|Goldie Tanjung Boi 1424
Melanotaenia goldiei|GoldieiMbuta 1397
Melanotaenia goldiei|Goldiei Bumi 1230
Melanotaenia goldiei|Goldiei Bumi 1228
Melanotaenia goldiei|Goldiei Bumi 1231
Melanotaenia goldiei|Goldiei Bumi 1226
Melanotaenia goldiei|Goldiei Bumi 1229
Melanotaenia goldiei|Goldiei Bumi 1227
Melanotaenia goldiei|Goldie Tanjung Boi 1427
Melanotaenia goldiei|Goldiei Avona1417
Melanotaenia goldiei|GoldieiMbuta 1396
Melanotaenia goldiei|Goldie Tanjung Boi 1426
Melanotaenia goldiei|Goldie Tanjung Boi 1423
Melanotaenia goldiei|Goldiei Avona1419
Melanotaenia goldiei|Goldiei Avona1418
Melanotaenia goldiei|Goldie Tanjung Boi 1422
Melanotaenia goldiei|Goldie Tanjung Boi 1425
Melanotaenia goldiei|Goldiei Avona1420
Melanotaenia goldiei|GoldieiMbuta 1400

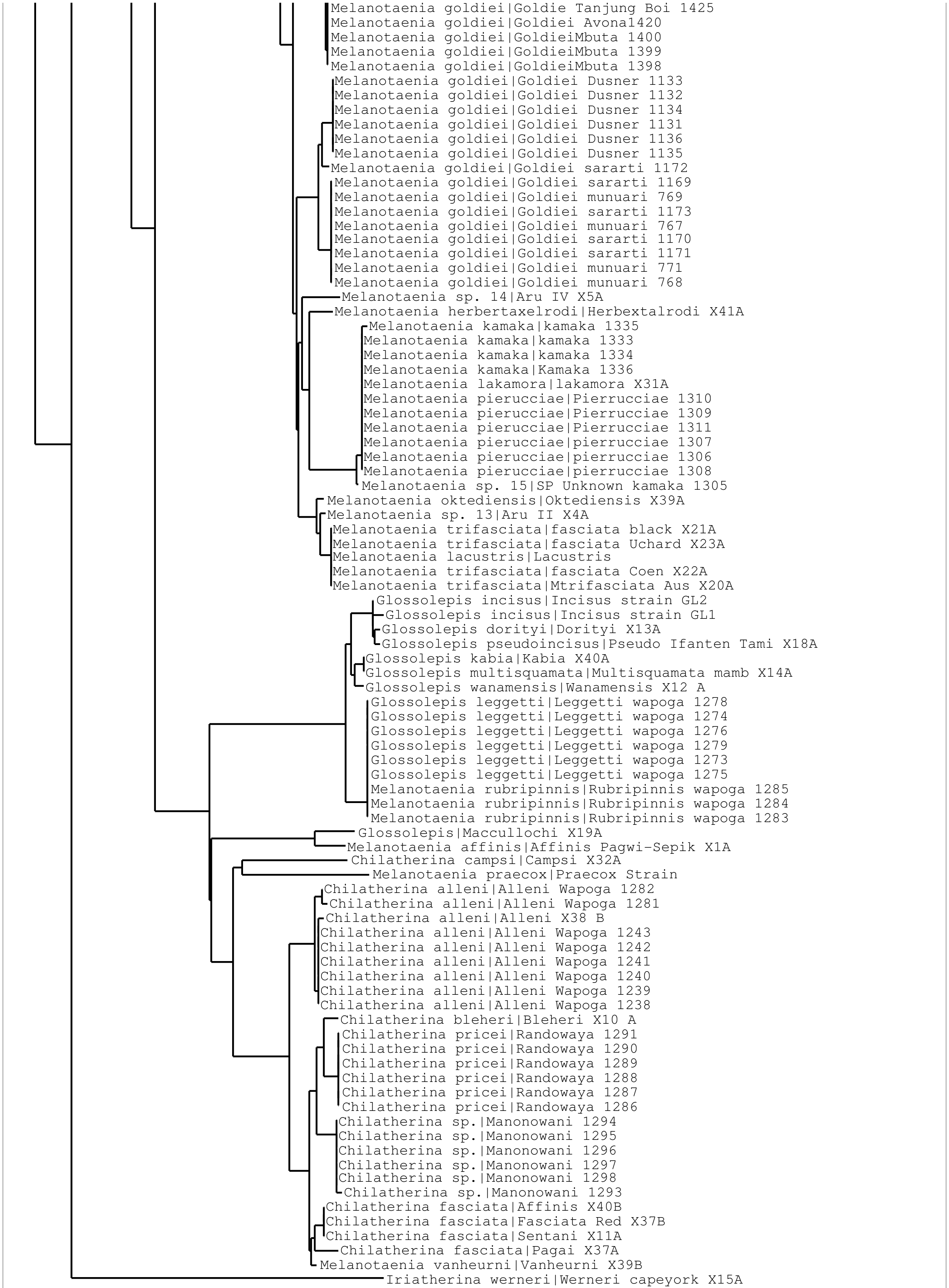
Melanotaenia goldiei|Goldie Tanjung Boi 1425
Melanotaenia goldiei|Goldiei Avona1420
Melanotaenia goldiei|GoldieiMbuta 1400
Melanotaenia goldiei|GoldieiMbuta 1399
Melanotaenia goldiei|GoldieiMbuta 1398
Melanotaenia goldiei|Goldiei Dusner 1133
Melanotaenia goldiei|Goldiei Dusner 1132
Melanotaenia goldiei|Goldiei Dusner 1134
Melanotaenia goldiei|Goldiei Dusner 1131
Melanotaenia goldiei|Goldiei Dusner 1136
Melanotaenia goldiei|Goldiei Dusner 1135
Melanotaenia goldiei|Goldiei sararti 1172
Melanotaenia goldiei|Goldiei sararti 1169
Melanotaenia goldiei|Goldiei munuari 769
Melanotaenia goldiei|Goldiei sararti 1173
Melanotaenia goldiei|Goldiei munuari 767
Melanotaenia goldiei|Goldiei sararti 1170
Melanotaenia goldiei|Goldiei sararti 1171
Melanotaenia goldiei|Goldiei munuari 771
Melanotaenia goldiei|Goldiei munuari 768
Melanotaenia sp. 14|Aru IV X5A
Melanotaenia herbertaxelrodi|Herbextalrodi X41A
Melanotaenia kamaka|kamaka 1335
Melanotaenia kamaka|kamaka 1333
Melanotaenia kamaka|kamaka 1334
Melanotaenia kamaka|Kamaka 1336
Melanotaenia lakamora|lakamora X31A
Melanotaenia pierucciae|Pierrucciae 1310
Melanotaenia pierucciae|Pierrucciae 1309
Melanotaenia pierucciae|Pierrucciae 1311
Melanotaenia pierucciae|pierrucciae 1307
Melanotaenia pierucciae|pierrucciae 1306
Melanotaenia pierucciae|pierrucciae 1308
Melanotaenia sp. 15|SP Unknown kamaka 1305
Melanotaenia oktediensis|Oktediensis X39A
Melanotaenia sp. 13|Aru II X4A
Melanotaenia trifasciata|fasciata black X21A
Melanotaenia trifasciata|fasciata Uchard X23A
Melanotaenia lacustris|Lacustris
Melanotaenia trifasciata|fasciata Coen X22A
Melanotaenia trifasciata|Mtrifasciata Aus X20A
Glossolepis incisus|Incisus strain GL2
Glossolepis incisus|Incisus strain GL1
Glossolepis dorityi|Dorityi X13A
Glossolepis pseudoincisus|Pseudo Ifanten Tami X18A
Glossolepis kabia|Kabia X40A
Glossolepis multisquamata|Multisquamata mamb X14A
Glossolepis wanamensis|Wanamensis X12 A
Glossolepis leggetti|Leggetti wapoga 1278
Glossolepis leggetti|Leggetti wapoga 1274
Glossolepis leggetti|Leggetti wapoga 1276
Glossolepis leggetti|Leggetti wapoga 1279
Glossolepis leggetti|Leggetti wapoga 1273
Glossolepis leggetti|Leggetti wapoga 1275
Melanotaenia rubripinnis|Rubripinnis wapoga 1285
Melanotaenia rubripinnis|Rubripinnis wapoga 1284
Melanotaenia rubripinnis|Rubripinnis wapoga 1283
Glossolepis|Maccullochi X19A
Melanotaenia affinis|Affinis Pagwi-Sepik X1A
Chilatherina campsi|Campsi X32A
Melanotaenia praecox|Praecox Strain
Chilatherina alleni|Alleni Wapoga 1282
Chilatherina alleni|Alleni Wapoga 1281
Chilatherina alleni|Alleni X38 B
Chilatherina alleni|Alleni Wapoga 1243
Chilatherina alleni|Alleni Wapoga 1242
Chilatherina alleni|Alleni Wapoga 1241
Chilatherina alleni|Alleni Wapoga 1240
Chilatherina alleni|Alleni Wapoga 1239
Chilatherina alleni|Alleni Wapoga 1238
Chilatherina bleheri|Bleheri X10 A
Chilatherina pricei|Randowaya 1291
Chilatherina pricei|Randowaya 1290
Chilatherina pricei|Randowaya 1289
Chilatherina pricei|Randowaya 1288
Chilatherina pricei|Randowaya 1287
Chilatherina pricei|Randowaya 1286
Chilatherina sp.|Manonowani 1294
Chilatherina sp.|Manonowani 1295
Chilatherina sp.|Manonowani 1296
Chilatherina sp.|Manonowani 1297
Chilatherina sp.|Manonowani 1298
Chilatherina sp.|Manonowani 1293
Chilatherina fasciata|Affinis X40B
Chilatherina fasciata|Fasciata Red X37B
Chilatherina fasciata|Sentani X11A
Chilatherina fasciata|Pagai X37A
Melanotaenia vanheurni|Vanheurni X39B
Iriatherina werneri|Werneri capeyork X15A
